# The Association between Nutritional Status and In-Hospital Mortality among Patients with Acute Coronary Syndrome—A Result of the Retrospective Nutritional Status Heart Study (NSHS)

**DOI:** 10.3390/nu12103091

**Published:** 2020-10-11

**Authors:** Michał Czapla, Piotr Karniej, Raúl Juárez-Vela, Katarzyna Łokieć

**Affiliations:** 1 Faculty of Health Sciences, Wroclaw Medical University, 51-618 Wroclaw, Poland; michal.czapla@umed.wroc.pl; 2 Department of Nursing, University of La Rioja, 26006 Logroño, Spain; raul.juarez@unirioja.es; 3 Department of Propaedeutic of Civilization Diseases, Medical University of Lodz, 90-251 Lodz, Poland; katarzyna.lokiec@umed.lodz.pl

**Keywords:** nutritional status, in-hospital mortality, acute coronary syndrome, NRS-2002, malnutrition

## Abstract

Background: Nutritional status is related to the prognosis and the length of hospitalization of individuals with myocardial infarction. This study aimed to assess the effects of nutritional status on in-hospital mortality in patients with acute coronary syndrome. Methods: We performed a retrospective study of 1623 medical records of patients admitted to the cardiology department of the University Clinical Hospital in Wroclaw (Poland) between 2017 and 2019. Results: It was found that, of those who died in the sample, 50% had a BMI within the normal range, 29% were in the overweight range and 18% were in the obese range. Patients who died had significantly more frequent occurrences of the following: Nutrition Risk Screening (NRS) ≥ 3 (20% vs. 6%; *p* < 0.001); heart failure (53% vs. 25%; *p* < 0.001); or a history of stroke (22% vs. 9%; *p* < 0.001), arterial hypertension (66% vs. 19%; *p* < 0001) or diabetes (41% vs. 19%; *p* < 0.001). Statistically significant differences were found when considering the type of infarction, diabetes or people with low-density lipoprotein greater than or equal to 70 mg/dL. Conclusions: This study shows that malnutrition correlates with an increased risk of death during hospitalization.

## 1. Introduction

Cardiovascular disease (CVD) is the leading cause of death in the European Union [1], where 15–33% of deaths occur due to CVD. The elimination of modifiable risk factors, such as an unhealthy lifestyle, could help prevent 80% of CVD cases [2]. However, making permanent changes, such as quitting smoking, maintaining a healthy weight, eating healthily and being physically active, has proven to be extremely difficult [3,4].

Malnutrition is not uncommon in patients with coronary artery disease, causing a worse clinical prognosis. According to Basta et al. [5], almost 55% of their study population with ST-segment elevation myocardial infarction (STEMI) were malnourished. Those individuals had a higher risk of dying from any cause when compared to those with a normal nutritional status. It is important to correctly assess the nutritional status of a patient who has experienced myocardial infarction, as early diagnosis and treatment may lower the risk of complications, rehospitalization and death [6]. Several tools have been developed for screening the nutritional status of patients on admission to a hospital. One of them is nutritional risk screening via the NRS-2002, a tool recommended by the European Society of Parenteral and Enteral Nutrition [7].

The treatment of patients with acute coronary syndrome (ACS) is a long-term and multistage process. It begins with the hospitalization of the patient and ends with changing eating habits, intensive cardiac rehabilitation and sometimes even implanting a heart electrostimulation device. The organization of health care for patients with ACS is an important factor that determines the chances of survival. Chung et al. [8] analyzed the ACS registers in Sweden and England between 2004 and 2010. The research results showed a significantly higher survival rate of patients with ACS in Sweden. Features of the health care system, such as the organization, treatment pathways and prevention programs, are not evaluated in randomized studies, but appear to have a significant impact on the morbidity and treatment of patients with ACS. All this information indicates that it is extremely important and necessary to educate patients on diet prophylaxis and lifestyle changes.

This study aimed to evaluate the effect of nutritional status on in-hospital mortality in patients with ACS.

## 2. Materials and Methods 

### 2.1. Study Design and Setting

We performed a retrospective study and analysis of medical records of patients admitted to the cardiology department of the University Clinical Hospital in Wroclaw (Poland) between January 2017 and August 2019 due to acute myocardial infarction (AMI) (ICD10: I21). The Strengthening the Reporting of Observational Studies in Epidemiology guidelines were followed.

### 2.2. Study Population

We analyzed all the patients who met the inclusion criteria (diagnosis of AMI, age ≥ 18 years old). A final group of 1623 patients’ medical records were analyzed. The analysis included data such as age, gender, patients’ body mass index (BMI) and laboratory results, such as total cholesterol (TC), high-density lipoprotein (HDL), low-density lipoprotein (LDL), triglycerides (TG), the type of myocardial infarction based on electrocardiography results, data on past and comorbid disease units and assessment of the nutritional status of the patient via the NRS-2002. 

### 2.3. Nutritional Screening

The NRS-2002 nutritional risk score consists of two components: nutritional status and severity of the disease. The NRS-2002 consists of two parts: impaired nutritional status and severity of the disease. The score of “impaired nutritional status” depends on three variables: BMI, weight loss (5% in the past 1, 2 or 3 months) and diet one week before hospitalization. The “severity of disease” part classified the patients according to the score of disease-related stress metabolism: absent, 0 points; mild, 1 point (e.g., hip fracture, diabetes, oncology); moderate, 2 points (e.g., major abdominal surgery, stroke); severe, 3 points (e.g., head injury, intensive care patients–acute physiology and chronic health evaluation, APACHE, < 10). The nutritional risk score is calculated by adding the score of impaired nutritional status (range from 0 to 3) and severity of disease (range from 0 to 3), and for patients 70 years and older, one additional point is added. The patient can have a total score from 0 to 7. Any patient with a total score ≥ 3 is considered to be at nutritional risk [9,10]. The NRS-2002 was calculated by a physician upon admission to the cardiology department. We used criteria from the Word Heart Organization to classify patients as underweight (BMI < 18.5), normal weight (BMI 18.5–24.9), pre-obese (BMI 25–29.9) and obese (BMI ≥ 30). 

### 2.4. Ethical Considerations

This single-center retrospective and observational study was approved by the independent Bioethics Committee of Wroclaw Medical University (decision no. KB–824/2019). The study was carried out in accordance with the tenets of the Declaration of Helsinki and recommendations for good clinical practice.

### 2.5. Statistical Analyses

Statistical analysis was performed using Statistica 13.1 (StatSoft, Inc., Tulsa, OK, USA). Arithmetic means and standard deviations were calculated for the measurable variables. All quantitative variables were tested with the Shapiro–Wilk test to determine the type of distribution. The between-group comparison was carried out using a *t*-test or Mann–Whitney U test (depending on the fulfillment of the assumptions). The comparison of the results of more than two groups was performed using one-way analysis of variance or the Kruskal–Wallis test (depending on the fulfillment of the assumptions). Survival analysis was conducted using the Kaplan–Meier method. The log-rank test was used to compare patient survival against selected clinical variables. The Cox proportional hazards model was used to assess the effects of qualitative and quantitative variables on survival. The model-building process was carried out using a progressive stepwise method, and a set of standard measures of goodness of fit (Akaike information criterion—AIC, R2 was used to evaluate the model. The analysis included categorical variables such as sex, heart failure (HF): Yes/No, Nutrition Risk Screening (NRS) <3 vs. ≥3, cerebral stroke (CS): Yes/No, myocardial infarction (MI): NSTEMI/STEMI), hypertension (HT): Yes/No, diabetes mellitus (DM): Yes/No, LDL: ≥70 vs. <70) and continuous variables such as age (years), body mass (kg), height (cm), BMI (kg/m^2^), TC (mg/dL), TG (mg/dL), HDL (mg/dL) and LDL (mg/dL). Due to the use of a univariate model, the variables BMI and LDL were analyzed as continuous and categorical variables. For the final multivariate model, variables (BMI and LDL) were selected depending on the better fit of the model based on the assessment of the goodness of fit (AIC). The results were considered statistically significant at *p* < 0.05.

## 3. Results

The characteristics of the entire group and the comparison of those characteristics between the survivors and those who died are presented in Table 1. A total of 1623 individuals were included in the analysis. The mean age was 69 ± 12 (the youngest patient was 31 and the oldest 98 years old). Due to a lack of data for some parameters, those numbers are smaller and are provided for each variable. In the group of patients who died, 56% were male; in the group of survivors, this percentage was higher, reaching 66% (*p* = 0.035). Statistically significant people who died had a BMI within a normal range. In the group of survivors, the respective distribution in terms of BMI was 28% (*n* = 268), 39% (*n* = 373) and 32% (*n* = 309). 

Patients who died had significantly more frequent occurrences of the following: NRS ≥ 3 (20% vs. 6%; *p* < 0.001); heart failure (53% vs. 25%; *p* < 0.001); or a history of stroke (22% vs. 9%; *p* < 0.001), arterial hypertension (66% vs. 19%; *p* < 0001) or diabetes (41% vs. 19%; *p* < 0.001). Furthermore, it was observed that a lower percentage of those who died had LDL greater than or equal to 70 mg/dL. Moreover, the deceased individuals were significantly older (x̅ = 75.4 vs. x̅ = 68.8 years; *p* < 0.001), with lower body weight (x̅ = 69.2 vs. x̅ = 79.4 kg; *p* < 0.001) and a lower BMI (x̅ = 25.5 vs. x̅ = 28.0 kg/m^2^; *p* = 0.004); they also had lower mean TC scores (x̅ = 149.8 vs. x̅ = 179.3 mg/dL; *p* < 0.001), HDL (x̅ = 36.4 vs. x̅ = 43.0 mg/dL; *p* < 0.001) or LDL (x̅ = 87.8 vs. x̅ = 108.2 mg/dL; *p* < 0.001) (Table 1).

A comparison of the assessed variables between the groups based on BMI are presented in Table 2. Four groups were distinguished: underweight, normal body weight, overweight and obese individuals. Statistically significant differences were found when taking into account the type of infarction, diabetes or people with LDL greater than or equal to 70 mg/dL. Non-ST-segment elevation (NSTEMI) myocardial infarction was more frequent in underweight patients when compared to STEMI (85%; *n* = 17). In the remaining groups, the percentage was as follows: normal BMI score—64% (*n* = 182), overweight score—63% (*n* = 243) and obese score—70% (*n* = 223). 

The occurrence of diabetes was more often observed in the obese group (30%; *n* = 95). A significantly higher percentage of people with LDL higher than or equal to 70 mg/dL was observed in the overweight group (86%; *n* = 313) (Table 2). Moreover, statistically significant differences were observed when taking into account parameters such as TC, TG, HDL and LDL. The highest mean scores of TC (x̅ = 186.4; *p* = 0.011) and LDL (x̅ = 114.8; *p* = 0.006) were reported in the overweight group, TG scores (x̅ = 164.7; *p* < 0.001) were the highest in the obese group and HDL scores (x̅ = 45.1; *p* < 0.001) were the highest in the group with a normal BMI (Table 2).

The comparison of the assessed parameters between groups based on the NRS score is presented in Table 3. Two groups were distinguished based on the NRS score: NRS < 3 and ≥3. Statistically significant differences were found when taking into account BMI, heart failure or people with LDL scores greater than or equal to 70 mg/dL. 

More frequently, a worse nutritional status was observed in underweight people or individuals with a normal BMI. Heart failure was statistically more common in patients with NRS ≥ 3. However, a smaller percentage of people with NRS ≥ 3 had an LDL score greater than or equal to 70 mg/dL. In the group with NRS < 3, the mean age was higher than in the group with NRS ≥ 3 (*p* < 0.001). Moreover, significantly lower results were observed in the group with NRS ≥ 3 in terms of body weight (*p* < 0.001), height (*p* = 0.024), BMI (*p* = 0.001), TC (*p* = 0.008) and LDL (*p* < 0.001). 

### 3.1. Survival Analysis

**Figure 1:**
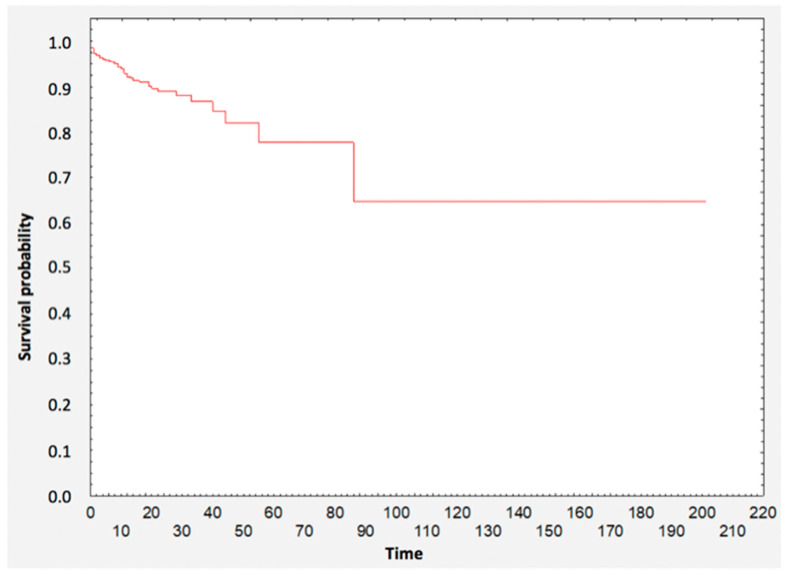
Analysis of survival for the entire group.

Patients’ survival analysis is presented with Kaplan–Meier survival curves (Figure 1). A group of 75% of patients survived the first 61 days. The total survival rate was 94% (*n* = 1518).

### 3.2. Survival Analysis—Group Comparisons

**Figure 2:**
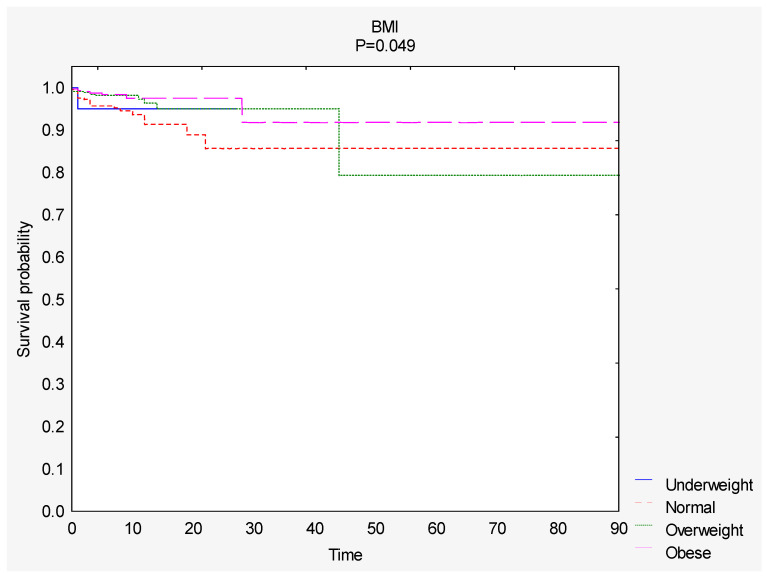
Comparison of survival curves depending on the BMI score. Abbreviations: BMI, body mass index.

Survival curves were compared based on the BMI score. The total survival rate was 96% (*n* = 970). A significantly greater survival rate was observed among people with a higher BMI (Figure 2). The total survival rate was 95% in the underweight group, 93% in the normal BMI group, 97% in the overweight group and 98% in the obese group (Table 4). 

**Figure 3:**
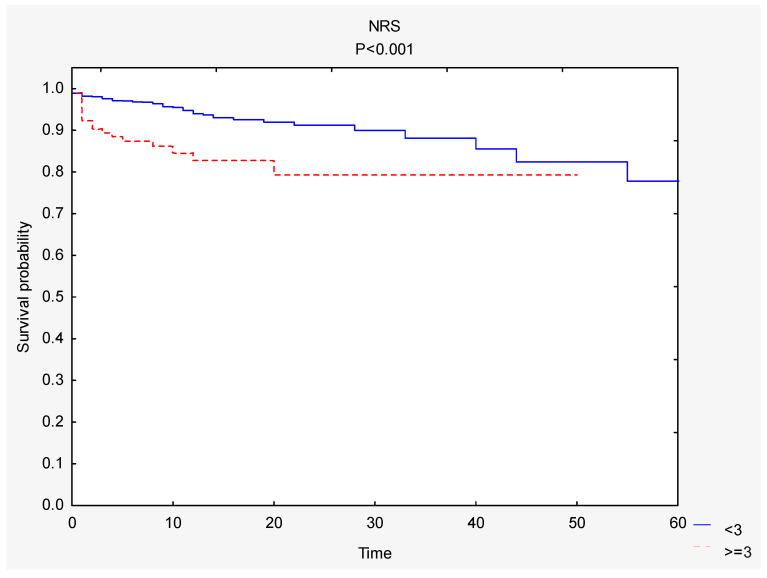
Comparison of survival curves depending on the NRS score. Abbreviations: NRS, nutritional risk screening.

Survival curves were compared based on the NRS scale. The total survival rate was 94% (*n* = 1352). A significantly greater survival rate was observed among people with a better nutritional status (Figure 3). In the NRS < 3 group, the total survival rate was 91%, whereas in the NRS ≥ 3 group, it was 84% (Table 4).

**Figure 4:**
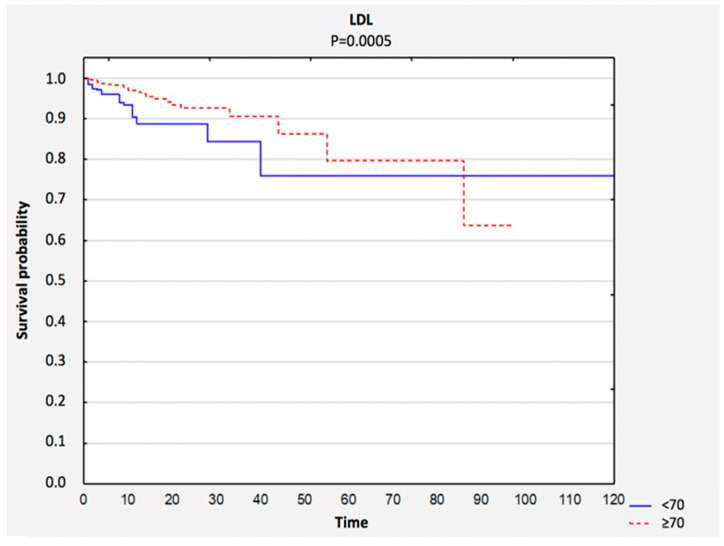
Comparison of survival curves depending on LDL result. Abbreviations: LDL, low-density lipoprotein.

Survival curves were compared based on the LDL level. The total survival rate was 96% (*n* = 1431). A significantly greater survival rate was observed among people with higher LDL levels (Figure 4). In the LDL < 70 group, the total survival rate was 91%, whereas in the LDL ≥ 70 group, it was 97% (Table 4). 

The evaluation of the impact of selected variables on mortality is presented in Table 5. It was observed that the risk of death increased when patients were older (hazard ratio, HR = 1.04; 1.03–1.06; *p* < 0.001), suffered from heart failure (HR = 2.15; 1.46–3.17; *p* < 0.001), arterial hypertension (HR = 7.38; 4.93–11.05; *p* < 0.001) or diabetes (HR = 2.59; 1.75–3.83; *p* < 0.001) or had a history of stroke (HR = 2.17; 1.36–3.47; *p* = 0.001), and their recovery status was worse (HR = 2.77; 1.62–4.75; *p* < 0.001). In contrast, the risk of death was reduced when patients had higher TC (HR = 0.99; 0.98–0.995; *p* = 0.001), HDL (HR = 0.96; 0.94–0.98; *p* < 0.001) and LDL scores (HR = 0.99; 0.98–0.997; *p* = 0.005) and when their body weight (HR = 0.97; 0.95–0.99; *p* < 0.001) or BMI (HR = 0.91; 0.85–0.97; *p* = 0.005) was higher and when they underwent NSTEMI infarction (HR = 0.60; 0.39–0.92; *p* = 0.019). 

Variables such as BMI (a categorized variable, as a quantitative BMI score was used), LDL (a categorized LDL score was used), body weight and height (scores correlated with BMI score) were not included in the multivariate model. The results that were confirmed in the multivariate model were heart failure (HR = 3.60; 1.70–10.50; *p* = 0.007), NRS > 3 (HR = 4.66; 1.60–16.61; *p* = 0.005), arterial hypertension (HR = 28.46; 12.70–112.60; *p* < 0.001), age (HR = 1.11; 1.06–1.16; *p* < 0.001), BMI (HR = 0.87; 0.77–0.94; *p* = 0.004), TG (HR = 1.01; 0.98–0.99; *p* = 0.007) and TC (HR = 0.99; 1.01–1.02; *p* = 0.017). 

## 4. Discussion

An increased risk of malnutrition is quite common among patients with CVD. At the same time, malnutrition is associated with a longer stay at a medical facility, increased number of hospitalizations, rehospitalization, the risk of treatment-related complications and even an increased risk of mortality. Therefore, it is a public health problem, as it increases the costs of the patient’s treatments [11,12,13]. The state of malnutrition among patients with AMI affects the number of complications and treatment outcomes [14]. In particular, the admission to hospital of older adults with AMI who are malnourished increases the risk of rehospitalization and death [15,16,17]. In this study, 20% of the malnourished patients died in the hospital. The risk of death was almost five times higher when the patient was at risk of malnutrition (HR = 4.66; *p* = 0.005). It was also observed that the risk of death increased with age (HR = 1.04; *p* < 0.001). This outcome is similar to the results in the research conducted by Lu et al. [18], where the in-hospital mortality of malnourished people was barely 20%. The researchers also reported that the risk of death increases with age (HR = 1.04; *p* = 0.002) and is 3.5 times higher (HR = 3.47; *p* < 0.001) in patients with identified malnutrition.

In the examined group of patients, a lower percentage of deaths with LDL ≥ 70 mg/dL (*p* < 0.001) was observed, and the risk of death was lower when patients obtained a higher LDL (HR = 0.99; *p* = 0.005), TC (HR = 0.99; *p* = 0.001) and HDL score (HR = 0.96; *p* < 0.001). In the primary prevention of CVD in high-risk patients, the European Society of Cardiology recommends LDL levels to be <70 mg/dL [19]. The “lipid paradox” has been reported in many clinical trials. Cho et al. [20] investigated this phenomenon in 9751 patients with AMI undergoing percutaneous coronary intervention. In-hospital mortality in this study was significantly higher in patients with LDL < 70 mg/dL. Depending on the model, the risk of death within 12 months after surgery was lower in patients with LDL at the level of 70–99 than in those with LDL < 70 (HR = 1.42 vs. 2.81).

Another study also showed that patients with AMI who had significantly lower TC, LDL and TG levels had a higher risk of dying within 30 days [21]. This phenomenon can be observed not only in patients with AMI, but also those with heart failure (HF) [22]. The lipid paradox was also confirmed in one of the largest cohort studies conducted in the United States by Reddy et al. [23]. The examined population in this study amounted to over 115,000 patients, and these researchers also reported that the decrease in LDL correlated with an increased risk of death during hospitalization. Furthermore, Xia et al. [24] concluded that the “TG paradox” may also occur in patients with coronary artery disease.

The cause of the lipid paradox is not fully understood. Patients may have extensive vasculitis after myocardial infarction, which may lead to this condition even with low lipid levels. It should also be taken into account that these individuals may have been treated with lipid-lowering drugs. Low levels of LDL can also be associated with poor nutritional status. 

Another factor that increases the risk of death during hospitalization is BMI. This study showed that a higher BMI result correlated with a reduced risk of death (HR = 0.85; *p* = 0.001). Patients with a lower BMI had NSTEMI-type infarctions more frequently (*p* = 0.041). Furthermore, STEMI-type AMI was associated with a 40% reduced risk of death (HR = 0.60; *p* = 0.019). A randomized trial conducted in Italy by De Luca et al. [25] showed that no relationship between BMI and survival or risk of complications was found in elderly patients with ACS. Song et al. [26], on the other hand, in a study conducted in China, reported that the in-hospital mortality of AMI patients decreased with an increase in BMI. In the cited study, among patients with BMI < 18.5, the risk of death doubled (HR = 2.01; *p* < 0.001), and among patients with BMI ≥ 29, the risk of death decreased by 50% (HR = 0.49; *p* < 0.001). Moreover, Holroyd et al. [26] found that the “obesity paradox” exists in patients from Great Britain who underwent percutaneous coronary intervention. Other researchers recognized the obesity paradox, but only in those older than 70 (HR = 1.69; *p* = 0.012) [27]. The phenomenon of the obesity paradox is somewhat controversial. Perhaps more intensive treatment of obese individuals contributes to this phenomenon.

A meta-analysis conducted by Niedzielska et al. [28] showed that obese people were 1 to 10 years younger than patients in the normal weight range. This may be why a physician can decide to intensify treatment and may explain the lower risk of hospitalization and mortality. The adipose tissue can also serve as a nutrient source when metabolism increases rapidly after AMI [29]. It should also be noted that in most studies, researchers used BMI to assess being overweight and obese. However, BMI is not a measure of body fat and does not differentiate between subcutaneous and visceral fat. 

The underlying factors of the obesity paradox remain uncertain, and this study may help with further research. However, it should be noted that the patient’s nutritional status is an important factor influencing the complications and risk of long-term mortality. It is important to improve the patient’s nutritional status [30]. In young women, being overweight or obese is associated with an increased risk of death due to AMI and CVD [30]. On the other hand, studies in elderly patients have shown that mortality risk of all-cause mortality was lowest in those who were classified as overweight according to BMI [31,32]. Weight reduction has more potential benefits in coronary artery disease (CAD) patients [33]. It is also worth mentioning that recent guidelines on the definition of malnutrition (e.g., Global Leadership Initiative on Malnutrition, GLIM adopt a different BMI threshold in people aged > 70 [34,35,36]. Scientists’ opinions around the world are divided, and the subject needs further research. 

### Study Limitations

The study had its limitations. Firstly, a small group of patients with an increased risk of malnutrition was included. They constituted 7% of the study group (*n* = 104). In some cases, no electrocardiography, NRS or BMI scores were included in the medical records. The documentation was also lacking information on the previous treatment of patients (e.g., with lipid-lowering drugs). Moreover, the patients’ body composition analyses were not conducted, and BMI results are not reliable indicators of being overweight or obese. Furthermore, the waist-to-hip ratio was not examined, and central obesity data based on waist circumference were not recorded. Lastly, due to limitations to access to personal data because of the anonymity of medical records, it was not possible to assess the long-term survival of patients after ACS.

## 5. Conclusions

Our study shows that malnutrition correlates with an increased risk of death during hospitalization. Higher levels of TC, LDL and HDL were associated with a lower risk of death, which may indicate a lipid paradox. A higher BMI score was associated with a significantly lower risk of death, which may indicate an obesity paradox. A lower risk of death during hospitalization was found among patients diagnosed with ACS NSTEMI. 

## Figures and Tables

**Table 1:** Characteristics of the group with a comparison of survivors and dead patients.

**Variables**	**Total**		**In-Hospital Mortality**					*p* *
			**Dead**			**Survivors**		
		***n***	**%**	***n***	**%**	***n***	**%**	
**Sex (*n* = 1623)**	**M**	1063	65.6	59	56.2	1006	66.3	0.035
BMI (*n* = 1007)	<18.5	20	2.0	1	2.6	19	2.0	0.024
	18.5–24.9	287	28.5	19	50.0	268	27.6	
	25.0–29.9	384	38.1	11	28.9	373	38.5	
	≥30	317	31.4	7	18.4	309	32.0	
NRS (*n* = 1435)	<3	1331	92.8	66	79.5	1265	93.6	<0.001
	≥3	104	7.2	17	20.5	87	6.4	
HF (*n* = 1623)	Yes	442	27.2	56	53.3	386	25.4	<0.001
CS (*n* = 1623)	Yes	153	9.4	23	21.9	130	8.6	<0.001
MI (*n* = 1623)	STEMI	494	30.4	36	34.3	458	30.2	0.005
	NSTEMI	1045	64.4	57	54.3	988	65.1	
	No info.	84	5.2	12	11.4	72	4.7	
HT (*n* = 1623)	Yes	356	21.9	69	65.7	287	18.9	<0.001
DM (*n* = 1623)	Yes	327	20.1	43	41.0	284	18.7	<0.001
LDL (*n* = 1493)	≥70	1184	79.3	37	59.7	1147	80.2	<0.001
**Variables**	**x̅**	**SD**	**x̅**	**SD**		**x̅**	**SD**	*p* **
Age (years) (*n* = 1623)	69	12	75.4	11.2		68.8	12.1	<0.001
Body weight (kg) (*n* = 989)	79.0	17.2	69.2	14.5		79.4	17.2	<0.001
Height (cm) (*n* = 986)	168.5	8.9	165.7	9.8		168.6	8.8	0.049
BMI (kg/m^2^) (*n* = 1007)	27.9	5.3	25.5	4.7		28.0	5.3	0.004
TC (mg/dL) (*n* = 1522)	178.1	53.3	149.8	55.7		179.3	52.9	<0.001
TG (mg/dL) (*n* = 1511)	141.3	90.4	126.9	69.2		143.1	91.2	0.16
HDL (mg/dL) (*n* = 1511)	42.7	11.9	36.4	13.7		43.0	11.7	<0.001
LDL (mg/dL) (*n* = 1493)	107.3	43.7	87.8	44.3		108.2	43.4	<0.001

Abbreviations: * χ^2^, test; ** *t*-test or Mann-Whitney U test; *n*, number of participants; M, males; x̅, mean; SD, standard deviation; *p*, level of significance; NRS, nutritional risk screening; HF, heart failure; CS, cerebral stroke; MI, myocardial infarction; HT, arterial hypertension; LDL, low-density lipoprotein; BMI, body mass index; TC, total cholesterol; TG, triglycerides; HDL, high-density lipoprotein; STEMI, ST-elevation myocardial infarction; NSTEMI, non ST-elevation myocardial infarction.

**Table 2:** The comparison of evaluated parameters with the ranges of BMI values.

**Variables**		**Total**		**BMI**								*p* *
				<18.5 *n* = 20		18.5–24.9 *n* = 287		25.0–29.9 *n* = 384		≥30 *n* = 317		
		*n*	%	*n*	%	*n*	%	*n*	%	*n*	%	
Sex	M	1065	65.6	10	50.0	187	65.2	266	69.3	202	63.7	0.18
HF	Yes	442	27.2	7	35.0	74	25.8	90	23.4	97	30.6	0.15
CS	Yes	153	9.4	0	0.0	34	11.8	32	8.3	21	6.6	0.06
MI	STEMI	494	30.4	2	10.0	87	30.3	128	33.6	79	25.2	0.041
	NSTEMI	1045	64.4	17	85.0	182	63.4	243	63.3	223	70.4	
	No info.	84	5.2	1	5.0	18	6.3	12	3.1	14	4.4	
HT	Yes	356	21.9	3	15.0	62	22.0	86	22.4	61	19.1	0.68
DM	Yes	327	20.1	3	15.0	38	13.2	62	16.2	95	30.1	<0.001
LDL	≥70	1184	79.3	12	63.2	214	78.7	313	85.5	235	78.6	0.013
**Variables**		**x̅**	**SD**	**x̅**	**SD**	**x̅**	**SD**	**x̅**	**SD**	**x̅**	**SD**	*p* **
Age (years)		69.2	12.1	72.4	10.5	70.1	12.7	68.9	12.2	67.6	11.6	0.058
Body weight (kg)		79.0	17.2	44.2	7.6	64.6	9.0	78.7	9.3	95.9	14.6	-
Height (cm)		168.5	8.9	166.7	9.9	168.0	8.7	169.3	8.6	168.1	9.2	0.13
BMI (kg/m^2^)		28.0	5.3	16.0	2.3	22.8	1.6	27.4	1.4	34.1	3.7	-
TC (mg/dL)		178.1	53.3	157.5	56.3	174.7	52.1	186.4	51.3	179.5	54.0	0.011
TG (mg/dL)		142.4	90.4	114.9	42.3	127.6	112.8	136.4	74.6	164.7	96.4	<0.001
HDL (mg/dL)		42.7	11.9	41.1	17.6	45.1	13.1	44.4	11.0	39.8	10.1	<0.001
LDL (mg/dL)		107.3	43.7	93.8	41.1	104.7	41.2	114.8	43.0	106.9	44.6	0.006

Abbreviations: * χ^2^, test; ** *t*-test or Mann-Whitney U test; *n*, number of participants; M, males; x̅, mean; SD, standard deviation; *p*, level of significance; NRS, nutritional risk screening; HF, heart failure; CS, cerebral stroke; MI, myocardial infarction; HT, arterial hypertension; LDL, low-density lipoprotein; BMI, body mass index; TC, total cholesterol; TG, triglycerides; HDL, high-density lipoprotein; STEMI, ST-elevation myocardial infarction; NSTEMI, non ST-elevation myocardial infarction.

**Table 3:** The comparison of evaluated parameters with the NRS.

**Variables**		**Total** ***n* = 1623**		**NRS < 3** ***n* = 1331**		**NRS ≥ 3** ***n* = 104**		*p* *
		***n***	**%**	***n***	**%**	***n***	**%**	
Sex	M	1065	65.6	879	66.0	59	56.7	0.06
BMI	<18.5	20	2.0	12	1.4	5	8.2	<0.001
	18.5–24.9	287	28.5	244	27.6	23	37.7	
	25.0–29.9	384	38.1	349	39.5	18	29.5	
	≥30	317	31.4	279	31.6	15	24.6	
HF	Yes	442	27.2	366	27.5	36	34.6	0.12
CS	Yes	153	9.4	126	9.5	14	13.5	0.19
MI	STEMI	494	30.4	418	31.4	22	21.2	0.06
	NSTEMI	1045	64.4	857	64.4	75	72.1	
	No info.	84	5.2	56	4.2	7	6.7	
HT	Yes	356	21.9	294	22.1	20	19.2	0.50
DM	Yes	327	20.1	256	19.9	23	22.1	0.56
LDL	≥70	1184	79.3	999	80.0	55	63.2	<0.001
**Variables**		**x̅**	**SD**	**x̅**	**SD**	**x̅**	**SD**	*p* **
Age (years)		69.2	12.1	68.9	12.0	76.8	11.7	<0.001
Body weight (kg)		79.0	17.2	79.6	16.8	71.3	20.7	<0.001
Height (cm)		168.5	8.9	168.8	8.8	164.5	8.3	<0.001
BMI (kg/m^2^)		28.0	5.3	28.1	5.2	26.2	6.1	0.006
TC (mg/dL)		178.1	53.3	179.6	53.2	156.3	52.9	<0.001
TG (mg/dL)		142.4	90.4	141.8	78.6	131.5	88.0	0.23
HDL (mg/dL)		42.7	11.9	42.6	11.6	43.2	14.4	0.61
Age (years)		107.3	43.7	108.9	43.9	85.4	36.5	<0.001

Abbreviations: * χ^2^, test; ** *t*-test or Mann-Whitney U test; *n*, number of participants; M, males; x̅, mean; SD, standard deviation; *p*, level of significance; NRS, nutritional risk screening; HF, heart failure; CS, cerebral stroke; MI, myocardial infarction; HT, arterial hypertension; LDL, low-density lipoprotein; BMI, body mass index; TC, total cholesterol; TG, triglycerides; HDL, high-density lipoprotein; STEMI, ST-elevation myocardial infarction; NSTEMI, non ST-elevation myocardial infarction.

**Table 4:** Descriptive statistics for survival time and number of deaths and survival depending on the BMI results, NRS scores and LDL scores.

Variables		Descriptive Statistics				
		Me	x̅	SD	*n*—Deaths	*n*—Survivors
**BMI**	<18.5	7.0	9.0	6.3	1	19
	18.5–24.9	7.0	10.7	10.5	19	268
	25.0–29.9	7.0	10.2	10.0	11	373
	≥30	7.0	10.5	14.2	7	310
**NRS**	≤3	7.0	10.7	11.8	66	1265
	>3	11.0	13.9	12.6	17	87
**LDL**	<70	9.0	12.2	14.9	25	284
	≥70	7.0	10.6	10.5	37	1147

Abbreviations: *n*, number of participants; Me, median; x̅, mean; SD, standard deviation; BMI, body mass index; NRS, nutritional risk screening; LDL, low-density lipoprotein.

**Table 5:** Evaluation of the influence of variables on mortality—regression proportional hazards model, single- and multi-factor model.

		Single-Factor Model				Multi-Factor Model			
Variables		*p*	HR	95%CI HR (Lower)	95%CI HR (Upper)	*p*	HR	95%CI HR (Lower)	95%CI HR (Upper)
**Sex**	M	0.051	0.68	0.46	1.01	-	-	-	-
**HF**	Yes	<0.001	2.15	1.46	3.17	0.002	4.22	1.70	10.50
**BMI**	<18.5	0.643	1.50	0.12	18.99	Not included in the model			
	25.0–29.9	0.389	0.44	0.21	0.93				
	≥30	0.155	0.35	0.15	0.82				
**NRS**	>3	<0.001	2.77	1.62	4.75	0.005	4.66	1.60	16.61
**CS**	Yes	0.001	2.17	1.36	3.47	-	-	-	-
**MI**	NSTEMI	0.019	0.60	0.39	0.92	-	-	-	-
**HT**	Yes	<0.001	7.38	4.93	11.05	<0.001	37.82	12.70	112.60
**DM**	Yes	<0.001	2.59	1.75	3.83	-	-	-	-
**LDL**	≥70	0.002	0.44	0.26	0.73	-	-	-	-
**Age (years)**		<0.001	1.04	1.03	1.06	<0.001	1.11	1.06	1.16
**Body weight (kg)**		0.001	0.97	0.95	0.99	Not included in the model			
**Height (cm)**		0.087	0.97	0.93	1.00	Not included in the model			
**BMI (kg/m^2^)**		0.005	0.91	0.85	0.97	0.001	0.85	0.77	0.94
**TC (mg/dL)**		0.001	0.99	0.98	0.995	0.017	0.99	0.98	0.99
**TG (mg/dL)**		0.22	0.99	0.99	1.00	0.007	1.02	1.01	1.02
**HDL (mg/dL)**		<0.001	0.96	0.94	0.98	-	-	-	-
**LDL (mg/dL)**		0.005	0.99	0.98	0.997	Not included in the model			

Abbreviations: *n*, number of participants; M, males; x̅, mean; SD, standard deviation; *p*, level of significance; HR, hazard ratio; CI, confidence interval; NRS, nutritional risk screening; HF, heart failure; CS, cerebral stroke; MI, myocardial infarction; HT, arterial hypertension; LDL, low-density lipoprotein; BMI, body mass index; TC, total cholesterol; TG, triglycerides; HDL, high-density lipoprotein; STEMI, ST-elevation myocardial infarction; NSTEMI, non ST-elevation myocardial infarction.
